# Responses of Rat Mesenchymal Stromal Cells to Nanocellulose with Different Functional Groups

**DOI:** 10.1021/acsabm.2c00794

**Published:** 2023-02-10

**Authors:** Ahmad Rashad, Martha Grøndahl, Ellinor Bævre Heggset, Kamal Mustafa, Kristin Syverud

**Affiliations:** † Center of Translational Oral Research (TOR), Department of Clinical Dentistry, University of Bergen, Bergen 5009, Norway; ‡ Department of Biotechnology and Food Science, Norwegian University of Science and Technology, Trondheim 7491, Norway; § RISE PFI, Trondheim 7491, Norway; ∥ Department of Chemical Engineering, Norwegian University of Science and Technology (NTNU), Trondheim 7491, Norway

**Keywords:** tissue engineering, wood-based cellulose nanofibrils, aldehyde functional group, protein adsorption, cell morphology, osteogenic differentiation

## Abstract

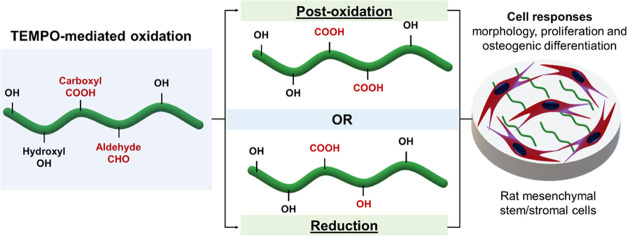

Cellulose nanofibrils (CNFs) are multiscale hydrophilic biocompatible polysaccharide materials derived from wood and plants. TEMPO-mediated oxidation of CNFs (TO-CNF) turns some of the primary hydroxyl groups to carboxylate and aldehyde groups. Unlike carboxylic functional groups, there is little or no information about the biological role of the aldehyde groups on the surface of wood-based CNFs. In this work, we replaced the aldehyde groups in the TO-CNF samples with carboxyl groups by another oxidation treatment (TO-O-CNF) or with primary alcohols with terminal hydroxyl groups by a reduction reaction (TO-R-CNF). Rat mesenchymal stem/stromal cells (MSCs) derived from bone marrow were seeded on polystyrene tissue culture plates (TCP) coated with CNFs with and without aldehyde groups. TCP and TCP coated with bacterial nanocellulose (BNC) were used as control groups. Protein adsorption measurements demonstrated that more proteins were adsorbed from cell culture media on all CNF surfaces compared to BNC. Live/dead and lactate dehydrogenase assays confirmed that all nanocellulose biomaterials supported excellent cell viability. Interestingly, TO-R-CNF samples, which have no aldehyde groups, showed better cell spreading than BNC and comparable results to TCP. Unlike TO-O-CNF surfaces, which have no aldehyde groups either, TO-R-CNF stimulated cells, in osteogenic medium, to have higher alkaline phosphatase activity and to form more biomineralization than TCP and TO-CNF groups. These findings indicate that the presence of aldehyde groups (280 ± 14 μmol/g) on the surface of TEMPO-oxidized CNFs might have little or no effect on attachment, proliferation, and osteogenic differentiation of MSCs.

## Introduction

Over the last decade, cellulose nanomaterials have emerged as promising biomaterials in the field of tissue engineering and regenerative medicine.^1−3^ Nanocelluloses have excellent physicochemical properties like high tensile strength, high specific surface area, and reactive surfaces combined with good cytocompatibility and renewability.^3,4^ The family of nanocelluloses includes three main types of cellulosic materials: the highly pure bacterial nanocellulose (BNC), the flexible long plant-based cellulose nanofibrils (CNFs), and the rigid short cellulose nanocrystals (CNCs).^1^ Compared to its plant-derived analogues, BNC has no lignin and hemicellulose because it is produced by Gram-negative bacteria as a neutral hydrogel-like biofilm. BNC has attractive physicochemical properties (such as density, Young’s modulus, and tensile strength) analogous to those of collagenous fibers in bone tissues.^5^ Morphologically, BNC fibers are comparable to collagenous nanofibers of native tissues, and therefore, BNC has shown great potential in tissue engineering applications.^6,7^ Like collagen scaffolds, BNC was reported to effectively promote adhesion, proliferation, and osteogenic differentiation of osteoblast-like cells.^8^ Still, biomaterials without microbial or animal-derived components are preferred immunologically. Considering this, great interest in plant-based CNFs has grown recently. Because of their large aspect ratio and flexibility, these nanofibrils can easily form hydrogels even at low concentrations.^2,9^ Lately, CNF hydrogels were used as tissue engineering scaffolds to support proliferation and differentiation of embryonic stem cells, liver cells, and mesenchymal stem/stromal cells (MSCs).^10−12^ CNFs, even at low concentrations, have favorable shear thinning properties because of their high aspect ratio, and therefore, they are usually added to bioinks used in bioprinting of stem/stromal cells to improve their rheological properties.^13−15^

Wood-based CNFs are usually produced by mechanical treatment coupled with some pretreatment strategies such as TEMPO (2,2,6,6-tetramethylpylperidine-l-oxyl) radical-mediated oxidation or carboxymethylation.^9,16^ These chemical pretreatment methods often change the surface properties of the extracted fibrils. Surface properties of biomaterials are known to regulate protein adsorption and subsequently cell responses.^17^ Surface functional groups, charges, wettability, topography, and stiffness are interconnected factors that can dictate cell adhesion, proliferation, and differentiation.^18−21^ In particular, functional groups have been found to be an important cue for osteogenic differentiation of stem cells.^22^ It was reported that surfaces with hydroxyl (OH) and amine (NH_2_) groups can upregulate osteoblast-specific gene expression, alkaline phosphatase (ALP) activity, and matrix mineralization compared with surfaces with carboxyl (COOH) and methyl (CH_3_) groups.^23^ Free neutral surfaces with CH_3_ and OH support less protein adsorption, cell spreading, and adhesion but greater chondrogenic differentiation of stem cells than charged surfaces with COOH and NH_2_.^18^ Moreover, human umbilical vein endothelial cells (HUVECs) were shown to adhere well to surfaces with COOH but not to surfaces with CH_3_. Arima and Iwata demonstrated that adhesion of HUVECs was improved by a CH_3_/OH surface (contact angle θ = 40°), whereas HeLa cells adhered best on CH_3_/OH and CH_3_/COOH surfaces (θ = 50°).^20^

Previously, we investigated the effect of two chemical pretreatments of CNFs, TEMPO-mediated oxidation (TO-CNF) and carboxymethylation (CM-CNF), on the adhesion of mouse fibroblasts. TO-CNF had aldehyde groups (211 ± 60 μmol/g) and carboxyl groups (764 ± 60 μmol/g). CM-CNF had carboxymethyl groups (346 ± 26 μmol/g) and less carboxyl groups (58 ± 1 μmol/g).^9^ Unlike TO-CNF, CM-CNF scaffolds adversely influenced the morphology of the cells and limited their spreading. We speculated that the poor adhesion is due to the carboxymethyl groups, while the good cell adhesion is because of the high density of the carboxyl groups. However, the role of the aldehyde group was not clear. There is little or no information about the biological role of the aldehyde groups on the surface of wood-based CNFs. The lack of knowledge of the interactions of the aldehyde groups with cells may become a barrier to developing effective CNF applications in tissue engineering. We hypothesized that the aldehyde groups may react with the amines of the adsorbed proteins on the surface of CNFs and subsequently improve cell responses. To test this hypothesis, in the current study we removed the aldehyde groups from the TEMPO-mediated oxidized CNFs by either oxidation to carboxyl groups or reduction to primary alcohols with terminal hydroxyl groups. To correlate protein adsorption to cellular responses, two-dimensional (2D) CNF-coated surfaces with and without aldehyde groups were first cultured with different protein solutions to determine the total protein adsorption. Second, rat bone marrow-derived stem/stromal cells (BMSCs) were cultured on the CNF surfaces, and their viability, morphology, proliferation, and osteogenic differentiation were evaluated. Overall, this study links the role of mixed functional surface groups (hydroxyl, carboxyl, and aldehyde) of wood-based nanocellulose to protein adsorption and subsequently to their biological performances for the use in bone regeneration applications.

## Experimental Section

### Preparation of CNFs with Different Functional Groups

TEMPO-mediated oxidized cellulose nanofibril gel-like samples (TO-CNF) were produced by chemical and mechanical treatments of fully bleached, never-dried softwood kraft pulp that was kindly donated by Södra Cell (Växjö, Sweden), according to a previously described method.^24^ Briefly, wood pulp (110 g) was suspended in water (8.25 L) containing 1.37 g of 2,2,6,6-tetramethylpiperidinyl-1-oxyl (TEMPO; Sigma-Aldrich) and 13.75 g of sodium bromide (NaBr). The pH was adjusted to 10.5 by adding 0.5 M NaOH. After that, 2.5 mmol NaClO/g dry cellulose was added slowly to the slurry, and pH was kept constant at 10.5. After 50 min, the pH was adjusted to 7 and methanol was added to remove TEMPO left in the slurry. Finally, the cellulose was washed thoroughly until the conductance of the filtrate was below 5 μS/cm. To prepare samples without aldehyde groups, selective oxidation and reduction of aldehyde groups were carried out. The TO-CNF was oxidized again (TO-O-CNF samples) with sodium chlorite (NaClO_2_) in water at pH 4–5 for 48 h at room temperature. TEMPO-oxidized cellulose (100 g, dry) was suspended in water (5 L) and mixed with NaClO_2_ (90.5 g) and 5 M acetic acid (1 L). The selective reduction of the TEMPO-oxidized CNFs (TO-R-CNF samples) was performed by adding 5 g of sodium borohydride (NaBH_4_) to the slurry at pH 8 for 48 h at room temperature. After washing, all nanocellulose samples were homogenized using a Rannie 15 type 12.56X homogenizer (APV, SPX Flow Technology). The bacterial nanocellulose (BNC) was purchased from JeNaCell (Germany) and used without any chemical modifications.

### Carboxylate and Aldehyde Contents

Total carboxylate content in all CNF samples was determined by the electric conductivity titration method. Briefly, 0.3 g of dry CNFs was added to water (450 mL) and 0.1 M NaCl (5 mL), and the pH was adjusted to 2.5–3.0 using 0.1 M HCl. The solution was stirred for 30 min before 0.05 M NaOH was added at a rate of 0.1 mL/min up to pH 11. During the titration, the conductivity of the solution was measured using the 856 Conductivity Module (Metrohm). The carboxylate content of cellulose was determined from the conductivity and pH curves. To determine the aldehyde content of the TO-CNF, samples were further oxidized with sodium chlorite. The carboxylate groups formed by this second oxidation were formed from aldehyde groups originally present in the TEMPO-oxidized CNFs.^24,25^ Furthermore, to confirm the absence of aldehyde groups in TO-O-CNF, TO-R-CNF, and BNC samples, 2,3,5-triphenyltetrazolium chloride (TTC) was used. Nanocellulose suspensions were mixed with 0.3 M KOH and 0.01 M TTC in water bath at 80 °C for 10 min. Oxidation of aldehydes leads to the reduction of TTC to the red compound 1,3,5-triphenyltetrazolium formazan (TTF) under alkaline conditions.

### Viscosity and Structural Characterization

For the viscosity measurements, a DV2TLV viscometer (Brookfield) was used along with Rheocalc software. Each CNF sample (0.4% CNF suspension) was evaluated in three parallels, and the viscosity of each parallel was measured from 0.1 to 100 rpm. For the nanostructures, 0.01% nanocellulose suspensions of each sample (25 μL) was added to clean the mica surface, left to dry in air, and then imaged with a Dimension ICON atomic force microscope (AFM) using NanoScope 9.4 Software (Bruker). Surface roughness (*R*_a_) was obtained from images (10 μm × 10 μm) using NanoScope Analysis software (version 1.9).

For the quantification of residual fiber content, a Fiber Tester 912 Plus (ABB AB/Lorentzen Wettre) was used. CNF suspensions were pumped through a flow cell where the fibers were photographed with a resolution of 4 μm. The images were further used to analyze the mean length and width of the fibers. To evaluate the microstructure of all cellulosic materials, tissue culture plates (24-well clear flat bottom polystyrene; NUNC, Denmark) were covered with cellulosic suspensions and stored at 37 °C for 24 h to dry. Films were analyzed using an optical microscope (Nikon Eclipse 80i, Tokyo, Japan) after staining with crystal violet.

### ζ-Potential, Contact Angle, and Protein Adsorption

The zeta (ζ) potential of the nanocellulose samples (0.08 g/L) was measured using a Zetasizer NANO ZSP (Malvern) apparatus with a Zetasizer software (version 7.11) cell culture medium (α-MEM; Life Technologies, Gibco, Carlsbad, CA) containing 10% FBS (pH 7.3). To study the effect of surface modifications on the wettability of the materials, the contact angle was measured with a DAT 1100 dynamic absorption tester (FIBRO system ab) at 23 °C and 50% relative humidity. Nanocellulose suspension (0.26%) was added to Petri dishes with a diameter of 5.6 cm and air-dried to make films for the contact angle measurement. A droplet of water (4 μL) was deposited on the specimen surface. A series of images were captured and analyzed by DAT3 software. The dynamic wetting (contact angle) was measured as a function of time between 0 and 175 s. To evaluate the effect of surface chemistry on protein adsorption, 500 μL of nanocellulose suspension (0.26%) of each sample was added to a 24-well plate and then incubated at 37 °C for 24 h. Uncoated TCP wells were used as controls. After that, nanocellulose-coated tissue culture plates were incubated with 500 μL of either single or complex bovine protein solutions for 4 h at 37 °C in a 5% CO_2_ humidified atmosphere. For single protein solutions, 5% bovine serum albumin (BSA; Sigma-Aldrich) in phosphate-buffered saline (PBS) and for complex protein solution, 100% fetal bovine serum (FBS; HyClone, GE Healthcare, Utah) solutions were used. To mimic cell culture conditions, 10% FBS in α-MEM was used. The wells were then washed with PBS (Life Technologies) to remove weakly adsorbed proteins and incubated with 2% sodium dodecyl sulfate (SDS, 500 mL/well) for 24 h to dissolve the adsorbed proteins. The total amount of proteins was measured by a commercial protein assay kit (Pierce BCA Protein Assay, Rockford, IL) according to manufacturer’s instructions.

### Cell Culture

The isolation of rat cells was approved by the Norwegian Animal Research Authority (local approval number 20,146,866). Bone marrow-derived mesenchymal stromal/stem cells were harvested from the femur of Lewis rats and characterized as described in a previous publication.^26^ Briefly, the metaphyseal ends of the femur were excised, and the marrow cavity was flushed with complete αMEM. The cells were resuspended in fresh αMEM medium containing 1% PS and 10% FBS and plated in cell culture flasks. After flow cytometry characterization, cells were negative for cluster of differentiation (CD) 34 and CD45 and positive for CD73 and CD90. Cells from passage 4 were used in the study. For cell seeding, wells of 24-well plates were covered with 500 μL of 0.26% nanocellulose suspension before drying over night at 37 °C to evaporate the water. The plates were sterilized under ultraviolet (UV) light for 1 h. Uncoated tissue culture plate (TCP) wells were used as controls. The cell seeding density was 5000 cell/cm^2^. In all experiments, fresh medium was supplied twice a week.

### Cytotoxicity and Morphological Assessments

To evaluate that the chemical pretreatments are cellularly safe and that the washing of the nanocellulose materials was sufficient to remove any harmful chemicals, an indirect cytotoxicity assay was conducted. Nanocellulose hydrogels were first sterilized using an autoclave and then incubated in αMEM (1 g/5 mL) at 37 °C with constant shaking (60 rpm) for 24 h, and then, the extracts were filtered (0.2 μm). As control samples, αMEM without hydrogels was kept at the same extraction conditions. Cells were plated and incubated in complete medium for 24 h to attach, and then, the media were replaced with extracts supplemented with 10% FBS. After 24 h, cell viability and mitochondrial activity were assessed utilizing live/dead and Alamar Blue assays (Invitrogen, Life Technologies). For live/dead stain, cells were incubated in a working solution containing ethidium homodimer-1 (stains dead cells red) and calcein AM (stains living cells green) for 30 min and then imaged with a fluorescence microscope (Nikon Eclipse Ti, Tokyo, Japan). For Alamar Blue, 50 μL of the reagent was added to each well and incubated for 4 h. The fluorescence was then measured utilizing a Varioskan LUX microplate reader (Thermo Fisher Scientific).

For direct cytotoxicity assessment, cells were cultured directly on nanocellulose-coated surfaces and TCP controls and then analyzed by live/dead staining after 24 h. To evaluate the toxicity of CNFs, lactate dehydrogenase (LDH) release was measured using a colorimetric kit (Abcam, Cambridge, U.K.). The enzymatic assay was conducted in accordance with the manufacturer’s instructions from the medium corresponding to the live/dead assay. For cell morphology, the cells cultured on nanocellulose-coated TCP were fixed with 4% paraformaldehyde after 4 and 24 h before being incubated in a solution of phalloidin-Atto488/PBS (Sigma-Aldrich; dilution 1:50) for 45 min in the dark at room temperature. At last, 4′,6-diamidino-2-phenylindole (DAPI; Sigma-Aldrich) in PBS (1:2000) was added for 5 min to label the nuclei. The cell geometry was visualized with a fluorescence microscope, and the cell surface area and maximum cell length were analyzed utilizing ImageJ software (1.46r).

### Cell Proliferation and Differentiation

To evaluate cell proliferation, double-stranded DNA (dsDNA) was quantified using a Quant-iT PicoGreen dsDNA Assay Kit (Invitrogen) in accordance with the manufacturer’s protocol. Briefly, after 1, 7, and 14 days of culture in growth or osteogenic medium (supplemented with β-glycerophosphate, ascorbic acid, and dexamethasone), cells were lysed with 0.1% Triton- X100 buffer before freezing (−80 °C). After two freeze–thaw cycles and sonication for 60 s, samples of each lysate (20 μL) were mixed with 180 μL of working solution in 96-well plates, and the fluorescence at 480/520 nm was measured with a microplate reader. To evaluate the early osteogenic differentiation of the MSCs, the release of alkaline phosphatase was measured from the same cell lysate after incubation with *p*-nitrophenyl phosphate (Sigma-Aldrich) for 15 min at room temperature. The absorbance was measured at 405 nm using a microplate reader, and the values were normalized to the DNA amount determined by the proliferation test. To study the late stages of osteogenic differentiation, Alizarin red S staining was performed after 21 days of culture in osteogenic medium. Samples were fixed in 4% PFA and then incubated with Alizarin red S (Sigma-Aldrich) solution for 15 min at room temperature. After washing and air-drying, images were taken with an optical microscope (Nikon, Tokyo, Japan). For quantification, the dye was extracted with 100 mM cetylpyridinium chloride (Sigma-Aldrich) at room temperature, and the absorbance was measured at 540 nm.

### Statistical Analysis

Statistical comparisons were conducted by one-way ANOVA with a Tukey’s post hoc multiple comparison using SPSS software (IBM). Data (*n* ≥ 3) are expressed as the mean ± standard deviation (SD). Differences were considered statistically significant at *p* ≤ 0.05.

## Results and Discussion

### Structure, Chemistry, and Viscosity of CNFs

**Figure 1:**
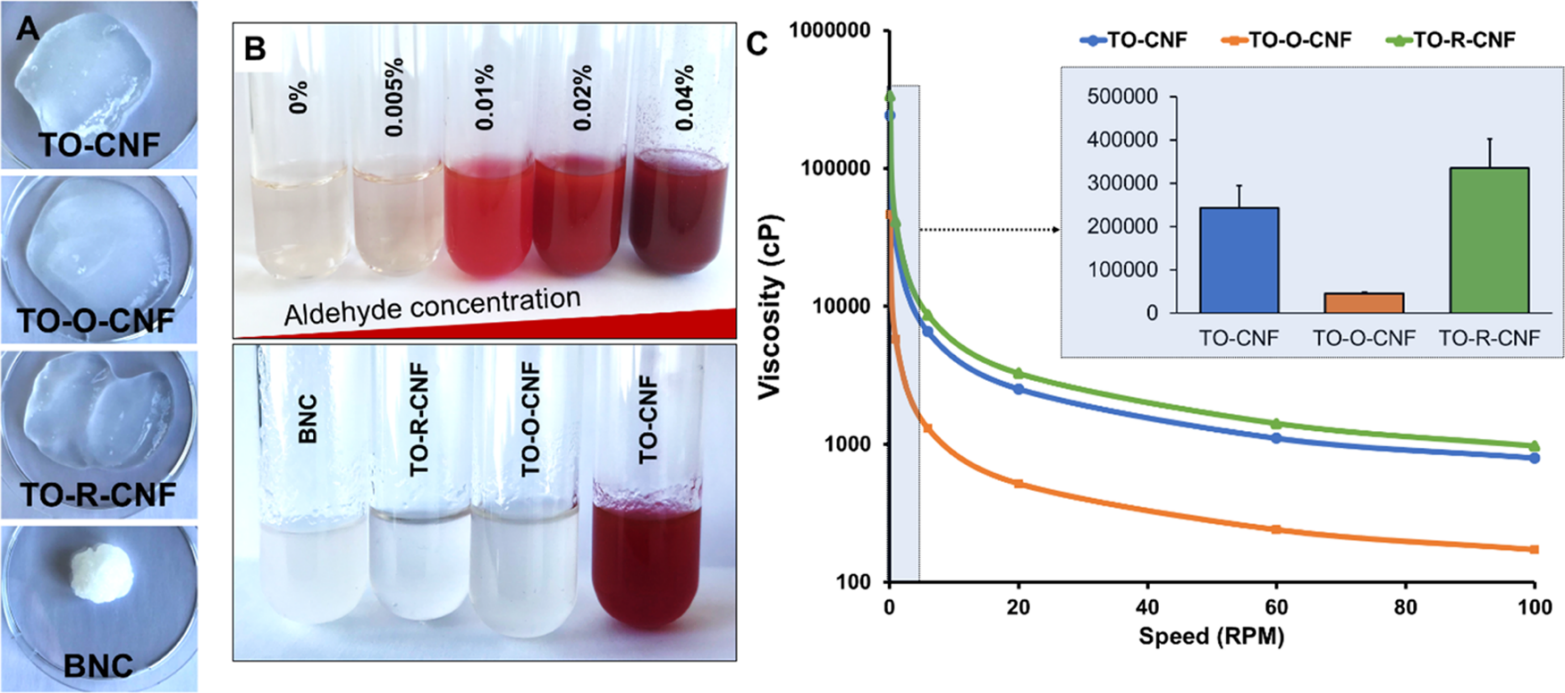
Preparation and characterization of nanocellulose hydrogels. (A) Macroscopic images of different CNF materials and BNC. (B) Aldehyde content of nanocellulose materials in reference to glucose concentrations (0 to 0.04% in water). (C) Change in viscosity of CNF suspensions as a function of shear rate (spindle speed).

In the present study, CNF hydrogels with and without aldehyde surface groups were produced by various chemical pretreatments prior to mechanical fibrillation of wood pulps. TEMPO-mediated oxidation, followed by either NaClO_2_ oxidation or NaBH_4_ reduction treatments was used to produce different viscous suspensions of CNFs at a low solid content (1%) as present in Figure 1A.

It has been reported that the C6 primary hydroxyl group of cellulose can be oxidized to aldehyde and carboxyl groups using TEMPO-mediated oxidation.^25,27^ As a result, negative charges are introduced to the surface of the fibers and facilitate their electrostatic repulsion.^16^ Therefore, this enables the disruption of the fibers into nano- and microfibrils.^28^ Our results confirmed the introduction of significant amounts of carboxyl (804 ± 3 μmol/g) and aldehyde (280 ± 14 μmol/g) groups by the TEMPO-mediated oxidation (Table 1). The second oxidation treatment of CNFs with NaClO_2_ converted the aldehyde groups to carboxyl groups, while the reduction reaction with NaBH_4_ changed the aldehyde groups to hydroxyl groups (alcohol). Consequently, TO-O-CNF had the highest carboxyl content (992 ± 24 μmol/g), while the TO-R-CNF had the least carboxyl content (675 ± 14 μmol/g). The detection of aldehyde groups in the different nanocellulose samples were investigated by color change after reacting with TTC. Oxidation of aldehydes leads to the reduction of TTC to the red compound of TTF.^27^ The amount of aldehyde groups was calculated from a calibration curve prepared with glucose solutions of different concentrations. Except for TO-CNF, all nanocellulose samples demonstrated absorbance values lower than the linear range of the standard curve. However, it is clear from the color that the aldehyde contents of TO-O-CNF, TO-R-CNF, and BNC are lower than that of TO-CNF and close to zero as shown in Figure 1B.

**Table 1:** Summary of the Surface Properties of Nanocelluloses

nanocellulose	aldehyde (μmol/g)	carboxyl (μmol/g)	roughness (*R*_a_) nm	contact angle	ζ-potential in medium (mV)
TO-CNF	280 ± 14	804 ± 3	46.1	48.6 ± 7.1	–10.0 ± 0.6
TO-O-CNF		992 ± 24	180.0	63.0 ± 6.6	–10.7 ± 0.5
TO-R-CNF		675 ± 14	43.0	63.8 ± 7.5	–9.7 ± 0.7
BNC			31.3	27.6 ± 7.9	–10.7 ± 0.6

Regarding the viscosity, CNFs form viscous hydrogels due to their hygroscopic nature, high aspect ratio, and high specific surface area, resulting in their strong interactions at low concentrations.^28^ The flow behavior of the prepared CNFs is presented in Figure 1C. All CNF hydrogels showed typical shear thinning behavior with a significant decrease in viscosity with the increase of the speed (shear rate). One of the main mechanisms dictating the mechanical strength of CNF hydrogels is their fiber entanglement.^29^ As shear increases, these entanglements are broken, and as a result, the fibers are separated and aligned with the flow, which explains the decrease in the viscosity. Moreover, Besbes et al.^56^ reported that the increase of carboxylic groups decreases the viscosity of CNF hydrogels, which is in line with our results. TO-O-CNF had the highest carboxyl content (992 ± 24 μmol/g) and the lowest viscosity value. The presence of such high levels of carboxyl groups on the surface of TO-O-CNF samples is likely to increase the electrostatic repulsion and reduce the extent of the interactions among CNFs, resulting in a lower viscosity of the suspension. Importantly, these results confirm not only the ability of the prepared CNF hydrogels to flow easily under high shear stress conditions but also the ability to tailor their flow behavior by chemical pretreatments. This is of great significance for the development of injectable hydrogels for drug delivery and for the development of bioinks for stem cell bioprinting in tissue engineering.^10,13^

### Morphological Assessment of CNFs

**Figure 2:**
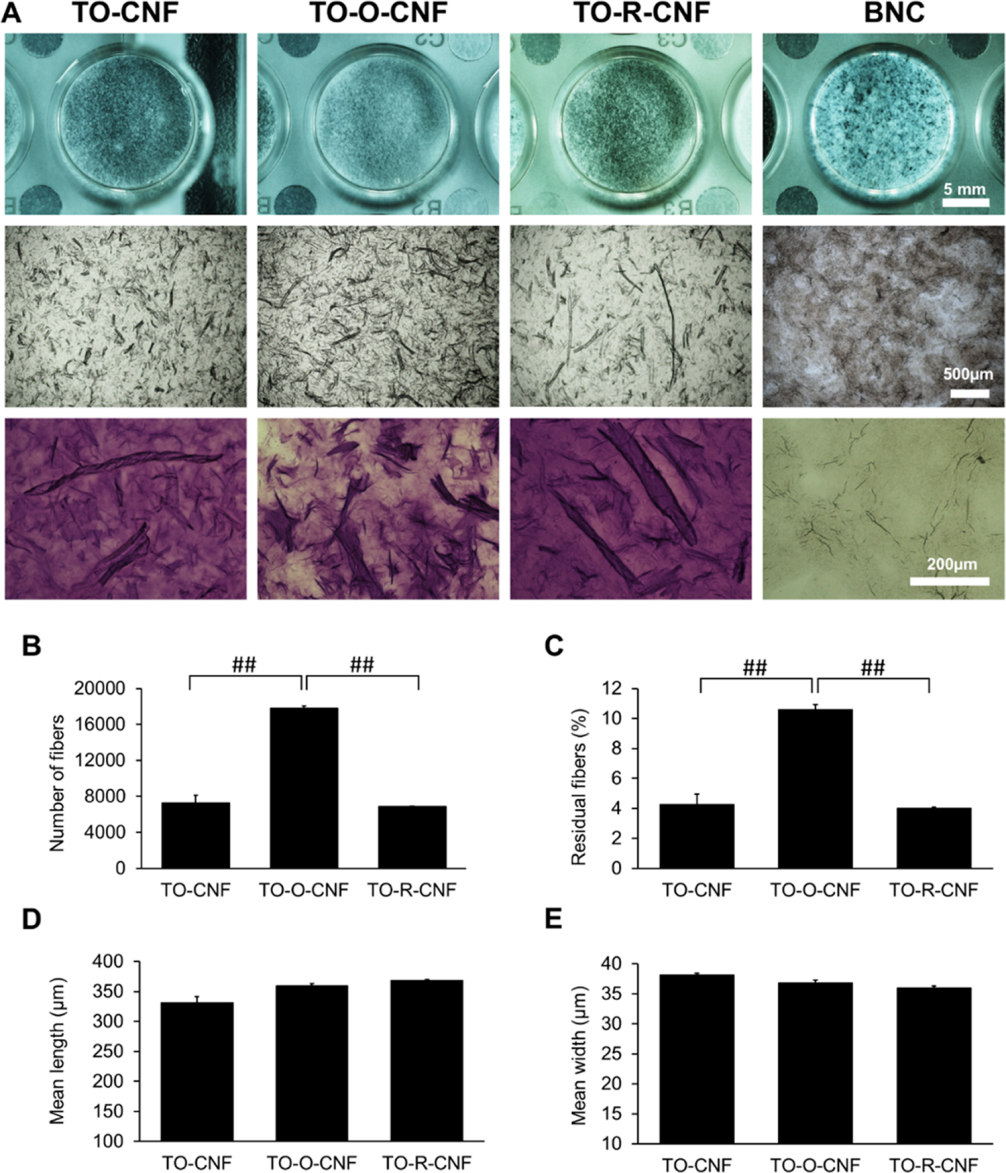
Morphological assessment and fiber analysis. (A) macroscopic (top) and microscopic images of nanocellulose before (middle) and after (bottom) staining with crystal violet. (B–E) Fiber analysis of CNF samples (*n* = 3). All values are expressed as mean ± SD (## *p* ≤ 0.001).

Fibrillation of cellulose by mechanical homogenization usually results in a highly heterogeneous mixture of fibers of different sizes with remaining large fibers and bundles of microfibrils.^30^ It appears that all CNF samples showed fibers of a few hundred micrometers in length, irrespective of the chemical pretreatment. In between the relatively large fibers, there was a dense ultrathin network observed by the crystal violet staining (Figure 2A).

**Figure 3:**
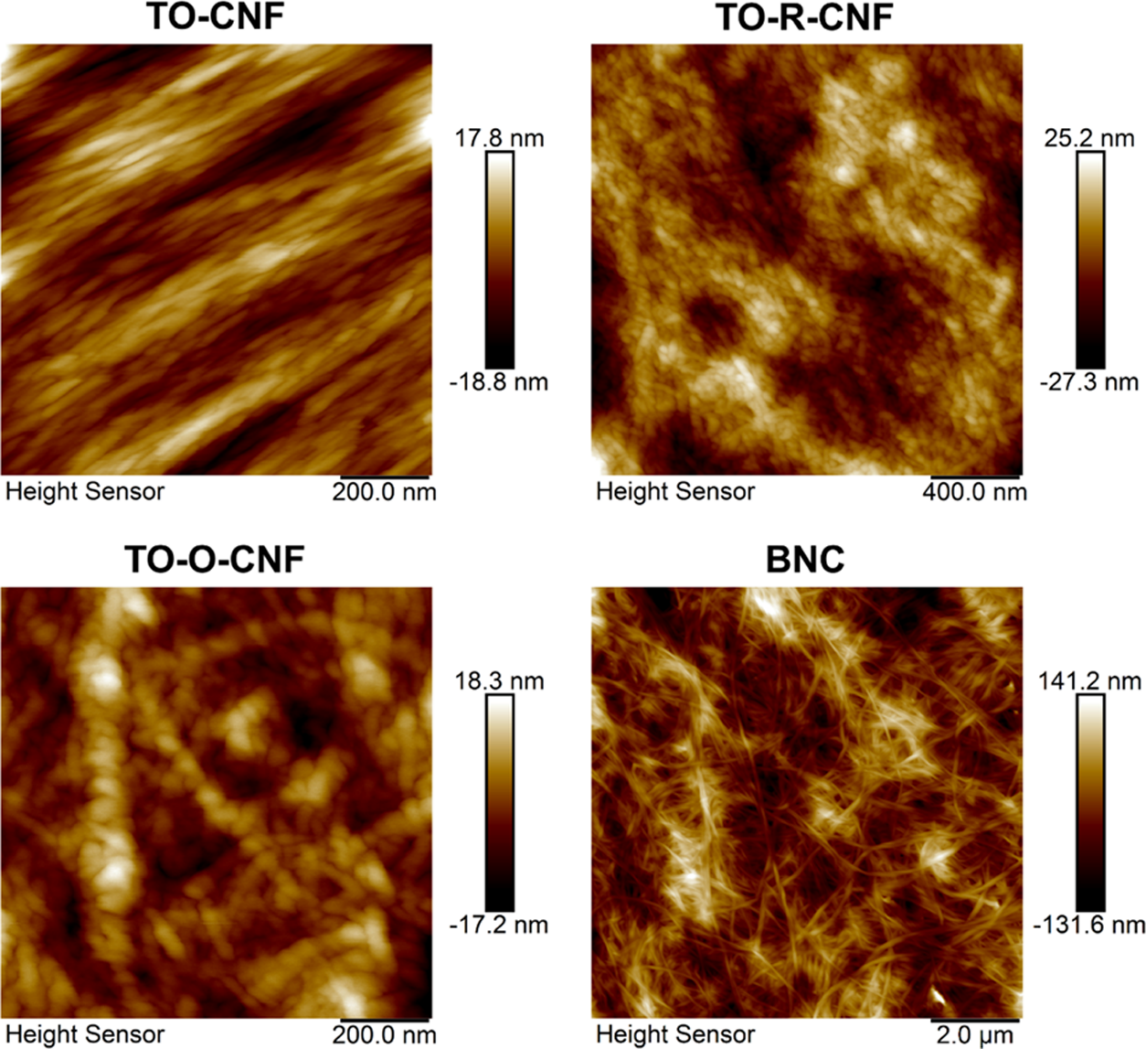
AFM images showing different nanoscale morphologies of CNFs and BNC materials.

The number of the microscale fibers are presented in Figure 2B. The TO-O-CNF showed higher residual fibers and higher number of the microfibers than other CNF samples (Figure 2B). Regardless of the chemical pretreatment, all CNF samples demonstrated a comparable fiber length and width (Figure 2D,E). To evaluate the morphology of the cellulose samples on the nanoscale, AFM analysis was conducted as shown in Figure 3. The arithmetic roughness average of the surface (*R*_a_) was extracted from AFM measurements. BNC demonstrated the smallest *R*_a_ (31 nm), while the TO-O-CNF group had the largest *R*_a_ (180 nm). These structural and morphological results are in line with the data obtained from the viscosity test. As the degree of fibrillation increases during the extraction process, the size of the fibrils generally decreases, and therefore, fibril–fibril interactions increase, resulting in a greater suspension viscosity.^31^ This can explain the low viscosity of TO-O-CNF due to the large size of these fibers and decreased fibril–fibril interactions.

### Wettability, Surface Charges, and Protein Adsorption

**Figure 4:**
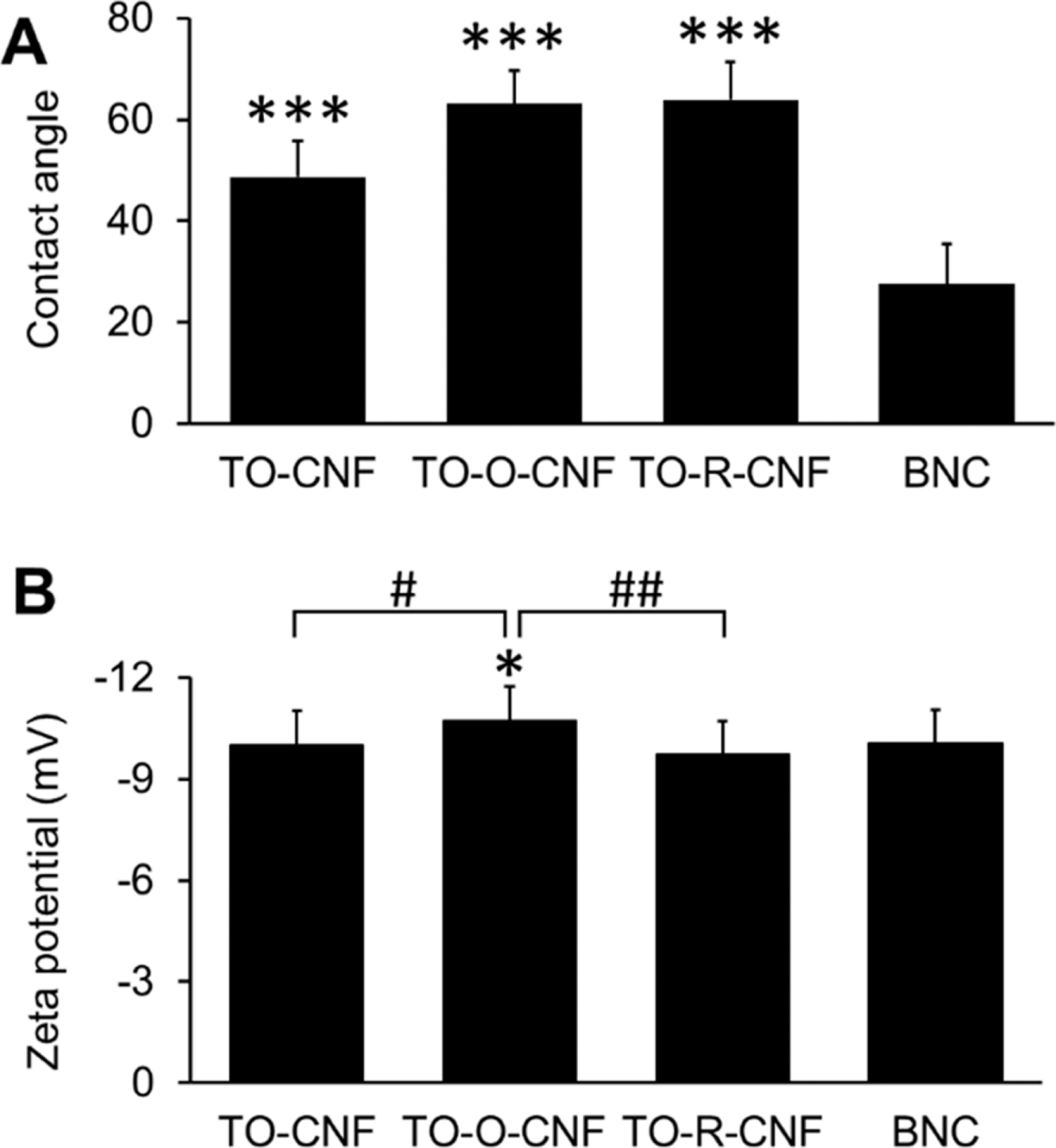
Water contact angle and ζ-potential measurements of different nanocellulose materials. (A) Water contact angle on 2D coated samples (*n* = 12). (B) ζ-Potential in growth medium with 10% FBS (*n* = 15). All values are expressed as mean ± SD. * Significant difference between BNC and any CNF groups (*, *p* ≤ 0.05; ***, *p* ≤ 0.001). # Significant difference between CNF groups (#, *p* ≤ 0.05; ##, *p* ≤ 0.01).

Figure 4A demonstrates the wettability of all nanocellulose materials, indicating their hydrophilic nature with contact angles less than 90°. The contact angle is known to be governed by chemical composition and topology of nanocellulose surfaces.^32^ Wu et al. reported that the wettability of CNF-based films correlated to the roughness values; the contact angle increased with the increase of surface roughness.^32^ In line with this, the contact angle of BNC in the current study was significantly smaller than those of all CNF groups due to their lower *R*_a_ (31 nm). Another possible explanation of the hydrophilicity of BNC is the presence of the high density of hydroxy polar groups on the surface.

To investigate the charge properties of the nanocellulose materials, ζ-potentials were measured in cell growth culture medium (pH = 7.4), and the respective data are included in Figure 4B. All nanocellulose samples demonstrated ζ-potential values (around −10 mV). However, the difference between TO-O-CNF and other groups was statistically significant because of the higher carboxylate density. In agreement with this result, Lopes et al. reported that the ζ-potential values of CNFs with different surface chemistries were negative for all materials (around −9 mV) when suspended in complete cell culture medium.^33^ This can be explained by the presence of FBS proteins in the cell culture medium, which mask the surface of the nanofibers and generate a new interface.

### Protein Adsorption

**Figure 5:**
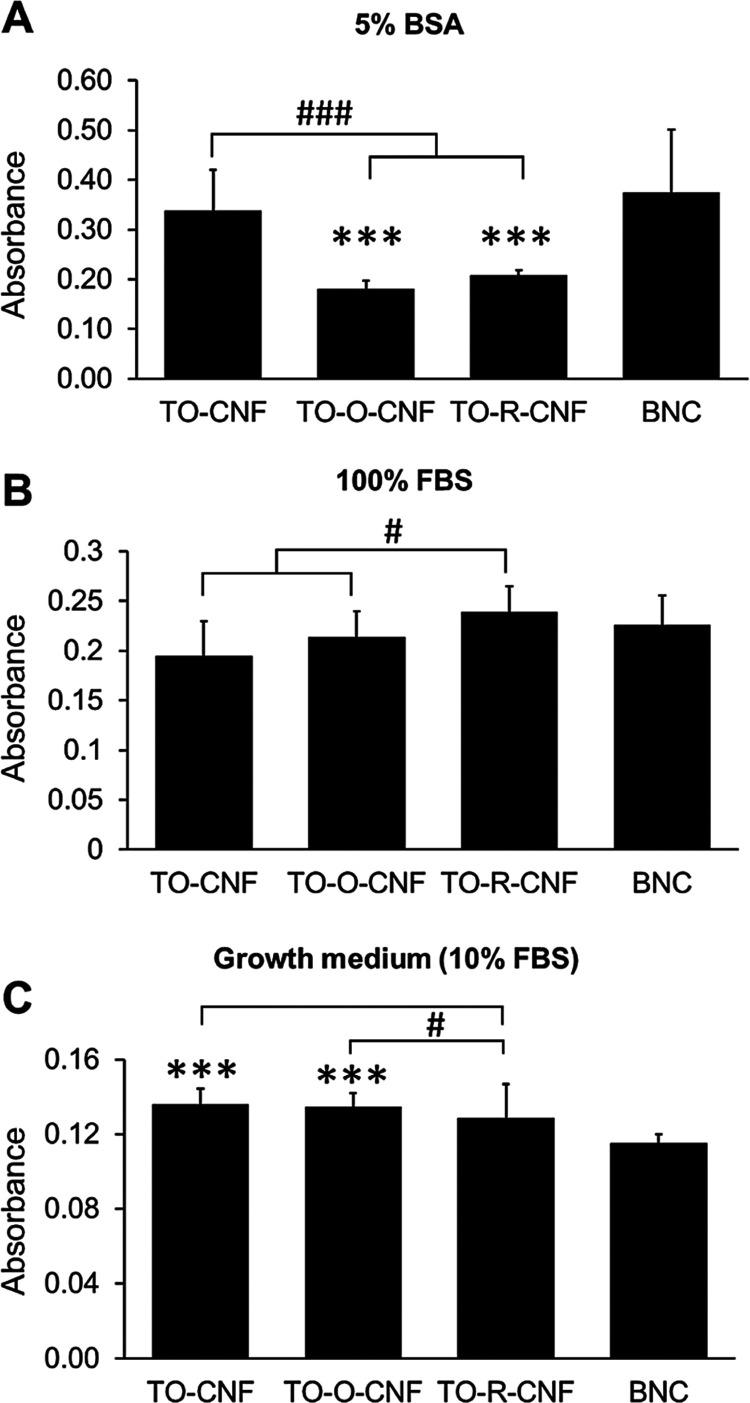
Protein adsorption onto different nanocellulose surfaces in (A) BSA solution, (B) FBS solution, (C) and 10% FBS solution. All values are expressed as mean ± SD (*n* = 5). * Significant difference between BNC and any CNF group (***, *p* ≤ 0.001). # Significant difference between CNF groups (#, *p* ≤ 0.05; ###, *p* ≤ 0.001).

Adsorption of proteins on any biomaterial surface is the first event that occurs in biological systems. Evaluating the adsorption of nonspecific proteins onto nanocellulose surfaces is important for their tissue engineering applications. Protein adsorption was greatly influenced by protein solution (type and concentration). When nanocellulose samples were soaked in 5% BSA solution, a significantly more protein amount was adsorbed on the surfaces of TO-CNF and BNC than that on the TO-O-CNF and TO-R-CNF (Figure 5A). In the case of 100% FBS, TO-R-CNF adsorbed significantly more proteins than TO-CNF and TO-O-CNF, as shown in Figure 5B. In contrast, a significantly greater protein amount was detected on the TO-CNF and TO-O-CNF than that on the TO-R-CNF and BNC (Figure 5C) when αMEM with 10% FBS was used. In agreement with that, Pajorova et al. reported different protein adsorption trends on CNF-coated surfaces when treated with BSA and FBS solutions.^34^ Adsorption of BSA, which is a negatively charged protein, was varied as a function of the strength of the electrostatic repulsion.^35^ Since CNFs have more negative charges, their surfaces adsorbed less BSA than BNC. Also, it has been reported that the protein adsorption on fibrous polymeric materials increases as the fiber diameter decreases.^36^ So, it is likely that BNC and TO-CNF, which have smaller diameters as confirmed by the AFM, adsorbed more amounts of BSA than TO-O-CNF and TO-R-CNF. Moreover, the aldehyde groups on TO-CNF can also explain the high BSA adsorption on TO-CNF as they can react with the amines of the adsorbed proteins via a Schiff base linkage. Yet, protein adsorption is a competitive phenomenon because any physiological environment involves multiprotein systems.^37^ Adsorption of a single protein on a biomaterial surface is affected by the presence of other proteins in the solution. This can explain the change in the adsorption behavior of nanocellulose surfaces in the case of FBS. Albumin, which is a small protein with a very high concentration in serum, tends to adsorb first, but it is partially replaced by larger proteins such as fibronectin and fibrinogen.^38^ Hasan et al. reported a linear increase in adsorbed FBS proteins with increase in surface hydrophobicity.^19^ This might explain why CNF groups (more hydrophobic surfaces) adsorbed more 10% FBS proteins than BNC samples. Generally, protein adsorption is a complex phenomenon controlled by biomaterial surface properties (e.g., roughness, wettability, and chemistry) and protein solution properties (e.g., pH, temperature, protein type, size, and concentration).^39^

### Indirect and Direct Cytotoxicity

**Figure 6:**
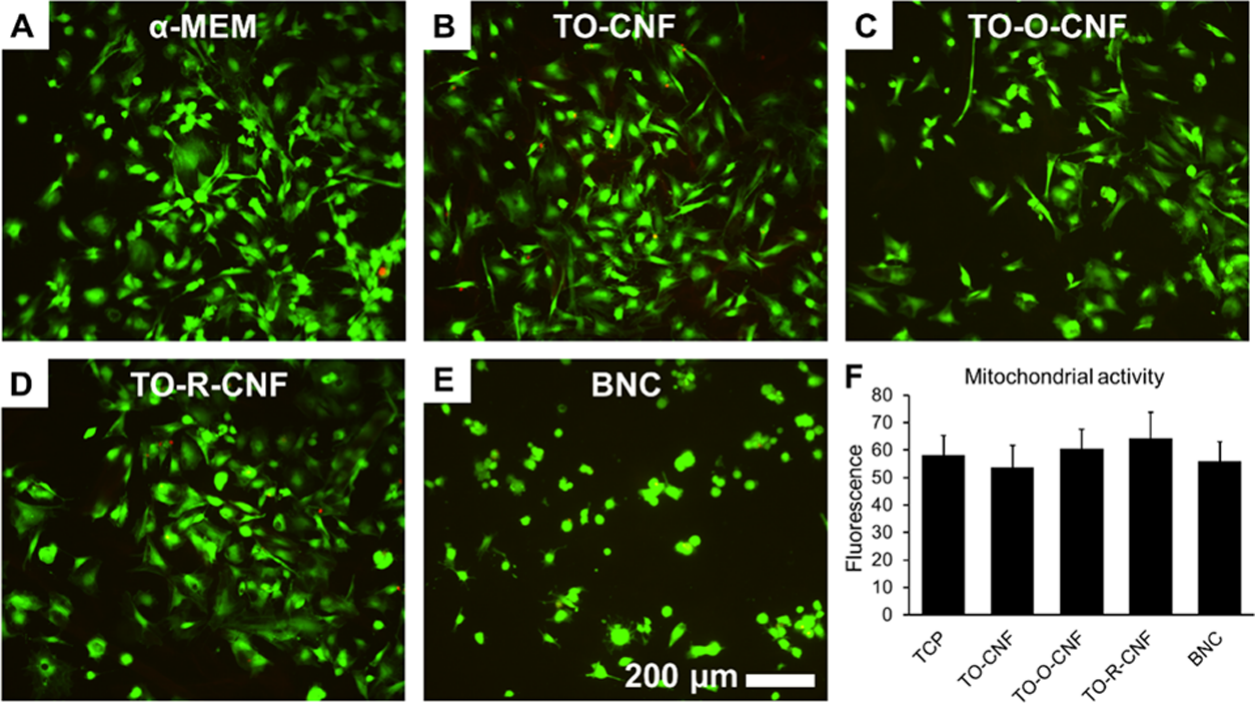
Indirect cytotoxicity assessment of rat MSCs treated with extracts of nanocelluloses. (A–E) Fluorescence images of live/dead stain. Calcein AM (green) represents live cells, and ethidium homodimer (red) represents dead cells. (F) Mitochondrial activity by the Alamar Blue assay. All values are expressed as mean ± SD (*n* = 4).

The potential release of any toxic products from the CNF materials after the chemical pretreatments was investigated by extract cytotoxicity test. The cellular response to the extracts of different nanocellulose materials was examined using the live/dead stain after 24 h (Figure 6). All nanocellulose groups demonstrated excellent cell viability, with most cells alive (green) and very few dead cells (red). The absence of cytotoxic effects of all nanocellulose extracts was confirmed by measurement of metabolic activity levels by the Alamar Blue assay (Figure 6F).

In our previous study, we demonstrated that, independently of the chemical treatments, CNF hydrogels showed no toxic effects against mouse fibroblasts.^9^ Similarly, Hua et al. reported that the cell viability of wood-based nanocellulose extracts was comparable to cell viability in regular culture medium after 24 h.^40^ Similarly, other reports demonstrated that the extracts of wood-based nanocellulose prepared by different chemical pretreatments had no negative effects on mitochondrial activity of different cells.^41,42^

**Figure 7:**
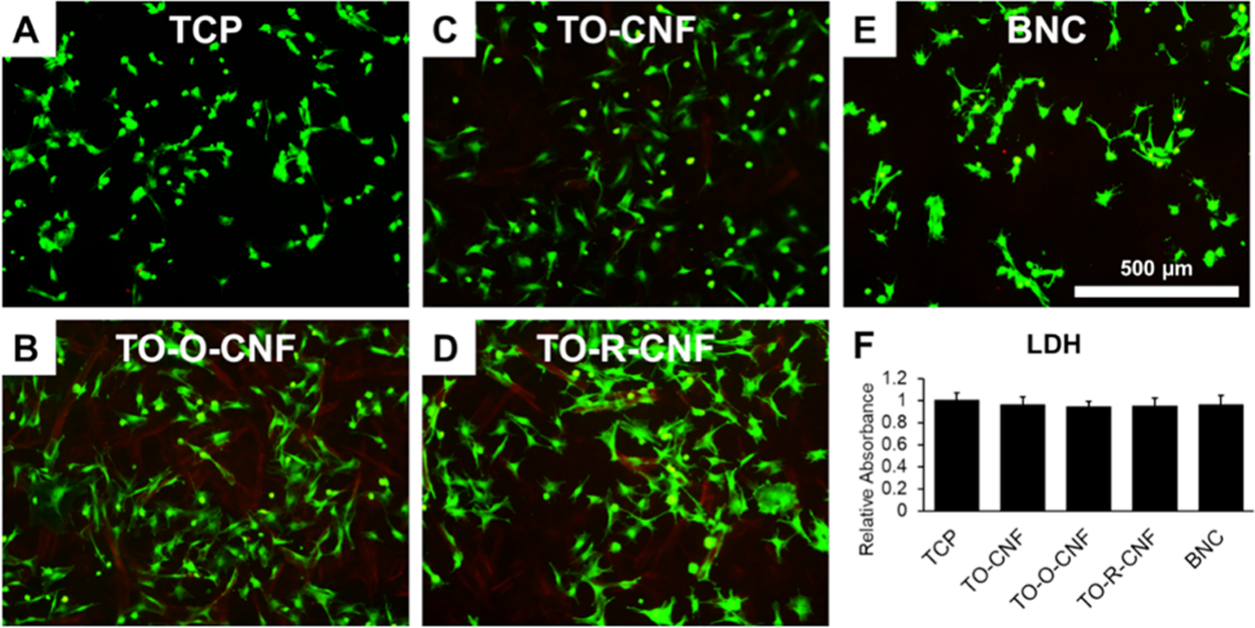
Direct cytotoxicity assessment of rat MSCs cultured on TCP, CNF, and BNC surfaces. (A–E) Fluorescence images of live/dead stain. (F) LDH assay. All values are expressed as mean ± SD (*n* = 4).

In the case of direct cytotoxicity assessment, cell viability was assessed utilizing live/dead stain and the LDH assay (Figure 7). Regardless of the surface chemistry, all cellulosic materials supported excellent cell viability after 24 h. The release of LDH from cells seeded on all nanocellulose samples was comparable to that of the cells cultured on the TCP controls. These findings agree with several reports, confirming the cytocompatibility of different nanocellulose materials.^9,10,43,44^

### Cell Adhesion and Morphology

**Figure 8:**
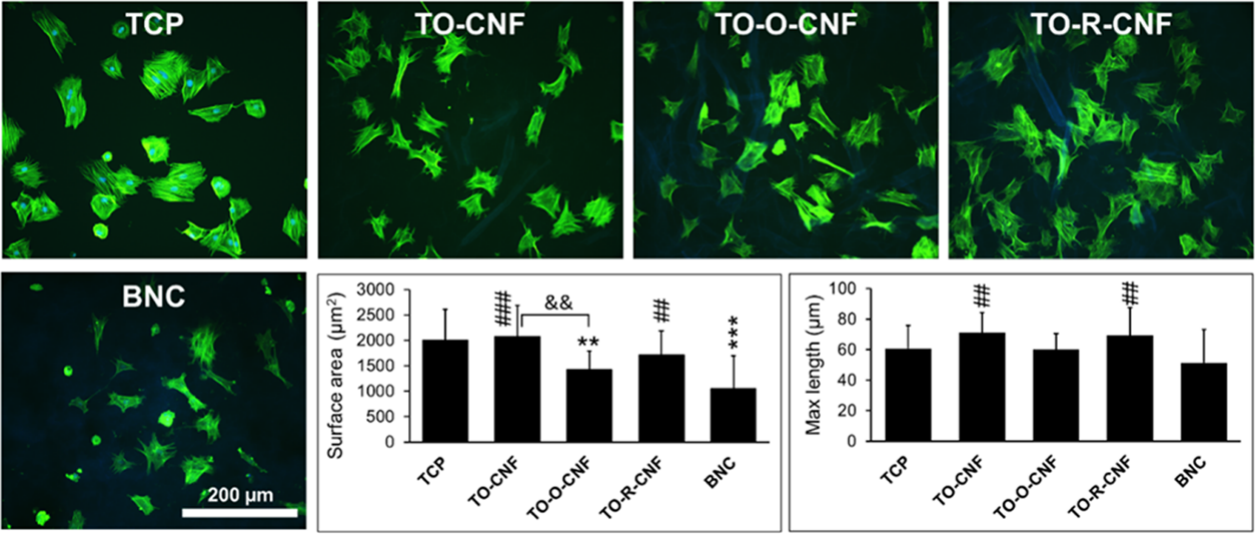
Cytoskeleton analysis of rat MSCs cultured on TCP, CNF, and BNC surfaces. Fluorescence microscopy images of the F-actin (green) and nuclei (blue). All values are expressed as mean ± SD (*n* = 20 cells). * Significant difference between TCP and other groups (**, *p* ≤ 0.01; ***, *p* ≤ 0.001). # Significant difference between CNF and BNC groups (##, *p* ≤ 0.01; ###, *p* ≤ 0.001). ^&^ Significant difference between CNF groups (&&, *p* ≤ 0.01).

The first events of cell–material interactions when cells are in direct contact with biomaterials are protein adsorption and cell adhesion. The cell morphology was visualized after fluorescence staining of F-actin in green and nuclei in blue after 4 h (data not shown) and 24 h (Figure 8).

After 4 h, cells on all surfaces showed round morphology. After 24 h, cells on TCP and TO-CNF (with OH, CHO, and COOH groups) surfaces demonstrated the largest surface areas, while cells on TO-O-CNF (with OH and COOH groups) and BNC (with the OH group) had the smallest surface areas. Replacement of aldehyde groups with hydroxyl groups in TO-R-CNF stimulated cells to spread with the surface area comparable to TCP and TO-CNF. These findings suggest that not only the type of the functional groups but also their amount is critical for directing cell response. Hua et al. demonstrated that human fibroblasts and osteoblast-like cells presented poor cell adhesion on nanocellulose without carboxylic surface groups. Specifically, they reported that carboxyl groups ≥260 μmol/g improved cell adhesion and morphology.^43^

In general, cells cultured on all CNF surfaces showed similar or slightly more elongated cells than cells cultured on TCP controls. Nevertheless, the maximum length of cells cultured on TO-CNF and TO-R-CNF surfaces was significantly higher than the length of cells on BNC. Regarding the surface chemistry, Cao et al. reported that mesenchymal stem cells exhibited more spreading morphology and larger surface areas on surfaces with carboxyl groups when compared to surfaces with hydroxyl groups.^18^ The ability of carboxyl-terminated CNFs to facilitate cell adhesion is due to their affinity to adsorb cell adhesion proteins such as fibronectin and vitronectin.^45^ In contrast, hydroxyl-terminated surfaces, such as BNC, exhibit poor protein adsorption due to their neutral charge and hydrophilic character.^18^ Our protein adsorption results demonstrated that the physicochemical properties of the BNC surface, including small *R*_a_ (31 nm) and small contact angle (27°), adsorbed more of the non-cell-adhesive BSA, which may explain the smaller cell surface area on the BNC surface.^34^ Moreover, CNF materials have a fibrous hierarchical structure of nano to microscale, which was reported to improve stem cell adhesion and osteogenesis.^46^ The absence of microfibers in BNC samples can be another possible explanation of the decreases in the cell area and cell length of the long axis.^47^ This could be attributed to the size of cell focal adhesions that are up to a few micrometers in size.^48^ Therefore, it is suggested that microscale topographical features may provide a stronger influence on focal adhesion formation than nanoscale features.^47^

Interestingly, TO-R-CNF, which has no aldehyde groups, showed comparable results to TCP and TO-CNF, indicating that the improved cell adhesion on TO-CNF samples is likely not related to the presence aldehyde groups. Compared to TO-CNF, TO-R-CNF has a larger contact angle (63.8 ± 7.5), which can explain the improved cell adhesion. Surfaces with a contact angle around 60° were reported to enhance cell adhesion.^19^ This speculation agreed well with other reports suggesting that cell adhesion gets optimum on surfaces with contact angles of 50–80°.^20,45^ However, the TO-O-CNF group has a similar contact angle (63.0 ± 6.6), but the cells cultured on its surface had significantly less surface areas than TO-CNF. Even though the physicochemical features of biomaterials are potent regulators of cell functions and can dictate their performance, it appears that the biological responses cannot be attributed to individual surface parameter alone.

### Cell Proliferation and Differentiation

**Figure 9:**
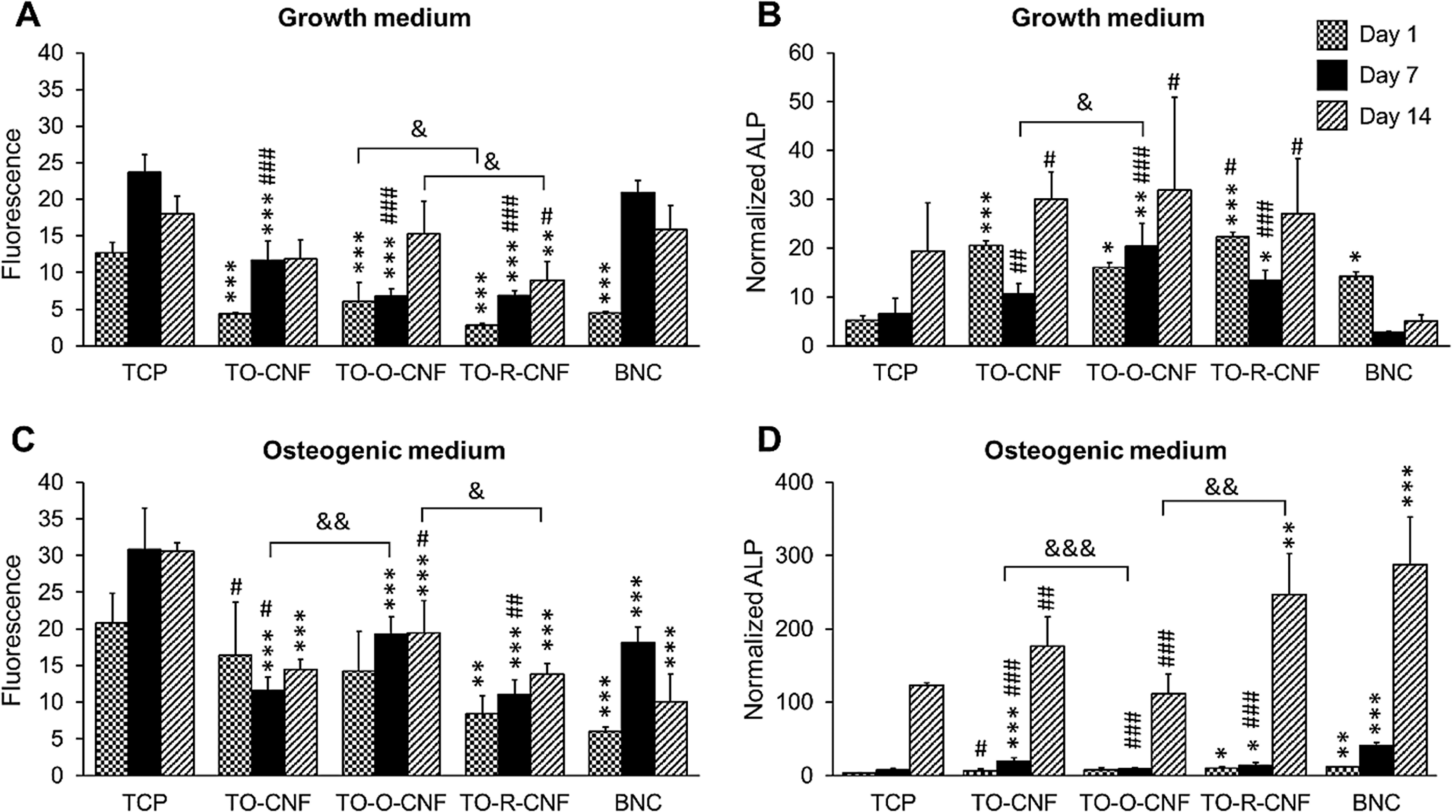
Cell proliferation and ALP activity of BMSCs cultured on TCP, CNFs, and BNC in (A, B) growth and (C, D) osteogenic media. All values are expressed as mean ± SD (*n* = 5). * Significant difference between TCP and other groups (*, *p* ≤ 0.05; **, *p* ≤ 0.01; ***, *p* ≤ 0.001). # Significant difference between CNF and BNC groups (##, *p* ≤ 0.01; ###, *p* ≤ 0.001). ^&^ Significant difference between CNF groups (^&&^, *p* ≤ 0.01).

Generally, all nanocellulose surfaces supported cell proliferation in growth medium (Figure 9A,C). At day 1, TCP demonstrated significantly higher cell number compared to all nanocellulose surfaces. At day 7, both TCP and BNC showed more cells than other CNF samples. At day 14, only TO-R-CNF exhibited significantly less cells than BNC and TCP. In the case of osteogenic medium (Figure 9C), the cells proliferated on all samples from day 1 to 14 except for TO-CNF. At days 7 and 14, the cell number on TCP was significantly more than all nanocellulose samples. Replacing the aldehyde groups with hydroxyl or carboxylic groups resulted in a significant impact on cell proliferation. At days 1 and 14 in growth medium, TO-O-CNF had more cells than TO-R-CNF. In osteogenic medium, a significantly higher cell number was found on TO-O-CNF when compared to TO-CNF at day 7 and TO-R-CNF at day 14. Altogether, both CNF and BNC surfaces supported cell proliferation, which agreed well with several reports.^9,44,46^

ALP, an early marker of osteogenic differentiation, was analyzed to assess the osteogenic potential of all nanocellulose surfaces (Figure 9B,D). After normalization to the total DNA amount, the ALP amount measured in growth medium was found to be elevated in all groups from day 1 to 14 except for BNC, which was significantly lower than those in all CNF materials. In addition, the early release of ALP from cells cultured on all nanocellulose materials at day 1 was significantly higher than TCP, confirming the role of surface roughness in initiating the osteogenic differentiation of MSCs.^46^ Kumar and colleagues showed that, in the absence of osteogenic supplements, polymeric nanofibers were able to drive the cells down the osteogenic lineage.^49^ In the case of the osteogenic medium, ALP production significantly increased from day 1 to 14 in all groups. The amount of ALP was always significantly higher in BNC and TO-R-CNF groups compared to that in TCP at any time point. Among the CNF groups, cells cultured on TO-O-CNF produced significantly less ALP than cells on the TO-CNF surface at day 7 and TO-R-CNF at day 14.

**Figure 10:**
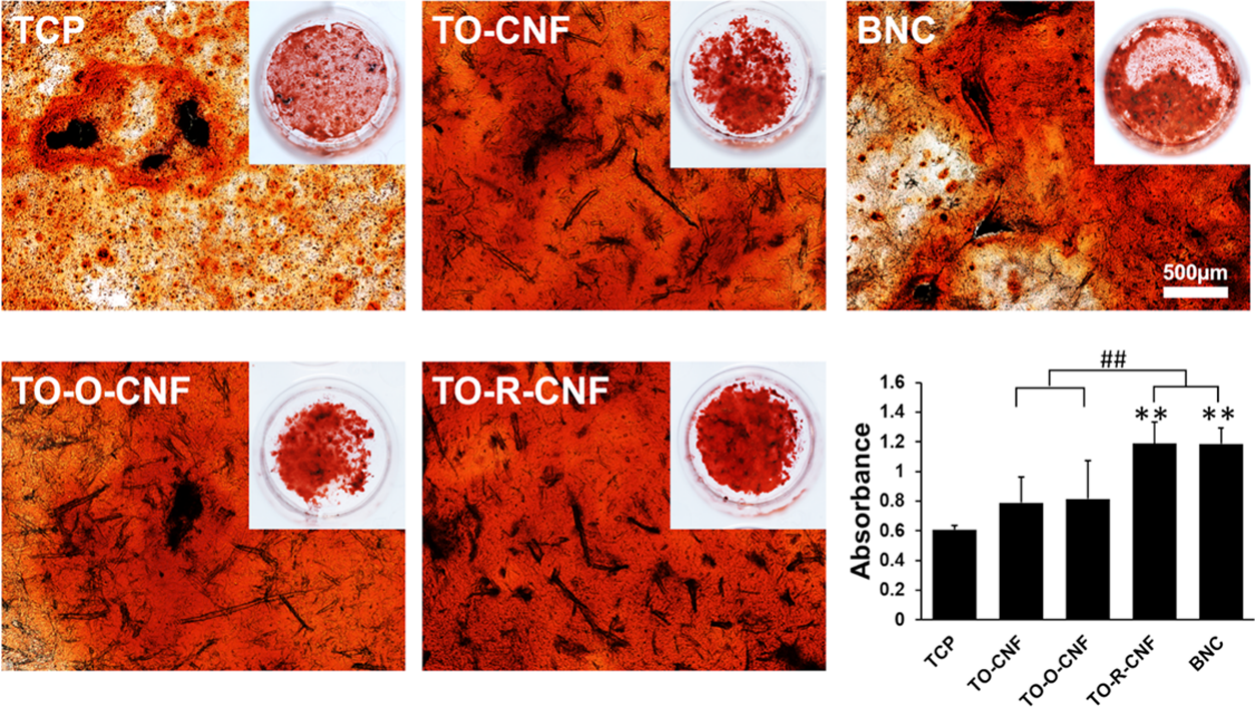
Mineralization assay by Alizarin red S at day 21. All values are expressed as mean ± SD (*n* = 5). * Significant difference between TCP and other groups (**, *p* ≤ 0.01). # Significant difference between CNF and BNC groups (##, *p* ≤ 0.01).

Moreover, the ability of BNC and TO-R-CNF to significantly support osteogenic differentiation of the cells, when cultured in osteogenic medium, was confirmed by Alizarin red S staining, as shown in Figure 10. In general, all nanocellulose supported more biomineralization than TCP after 21 days. However, this difference was statistically significant only in the TO-R-CNF and BNC groups. Differentiation of MSCs is known to be regulated by both physical and chemical surface properties of biomaterials. MSCs interact with the surrounding extracellular matrix or biomaterial surfaces through integrin receptors that sense and link the cell to its surrounding physical environment.^50^ Integrins bind to their ligand as a heterodimer of two α and β subunits. There are several α (α) and β (β) subunits indicating that, through their different possible combinations, several cellular signaling pathways can be activated.^50^

At the level of surface chemistry, biomaterials with carboxylic and hydroxyl groups have been shown to promote and maintain chondrogenesis but did not support osteogenesis.^51^ In contrast, other reports suggested that OH-terminated surfaces supported significantly more osteoblastic gene expression, ALP activity, and matrix mineralization than COOH-terminated surfaces.^23^ This can explain the effect of BNC, which has more OH-terminated surfaces. Interestingly, TO-R-CNF has both hydroxyl and carboxylic groups but showed more biomineralization than other groups. This finding indicates that the osteogenic differentiation might be related not only to surface chemistry. Both BNC and TO-R-CNF demonstrated the smallest *R*_a_ values (31 and 43 nm, respectively), which may explain their promoted mineralization results. In agreement with this speculation, Khang et al. suggested that the nanoroughness is influential in promoting osteoblast differentiation through integrin activation and expression of cyclins.^52^ Faia-Torres et al. confirmed that changes in polymer roughness (*R*_a_) may affect the commitment and degree of MSC osteogenic differentiation.^53^ Moreover, surface chemistry, wettability, and surface charges were reported to affect cell adhesion and differentiation not only by the adsorption of adhesive proteins (e.g., fibronectin and vitronectin) but also by changing their conformations.^18,51,54^ For example, it was shown that the biomineralization was promoted on negatively charged substrates with carboxyl groups when the integrin β3 subunit was blocked.^55^ While it is hypothesized that the binding of αvβ1 integrin promotes osteogenic differentiation, αvβ3 integrin, on the other hand, suppresses bone mineralization.^54^ The negative charges from COOH may cause a conformational change in the adsorbed adhesive proteins, which promotes the binding of both α5β1 and α5β3 integrins. Such conformational changes may alter the integrin binding affinity and subsequently activate different signaling pathways that leads to different stem cell differentiation results.^54^ However, most of the data published to define the relationship between surface chemistry and MSC differentiation is based on single monolayer or hybrid systems with one or two functional groups involved.^19,45,51^ Indeed, the multiscale fibrillar nature of the cellulosic materials with their mixed functional groups and different wettability and charges makes it difficult to identify a direct correlation between their chemistry and cell responses. Importantly, the interaction between cells with a biomaterial surface is a complex bidirectional process, and further investigations should be considered.

## Conclusions

In this study, the role of aldehyde functional groups of TEMPO-mediated oxidized CNFs was investigated in vitro with rat bone marrow mesenchymal stem/stromal cells, in terms of morphology, proliferation, and osteogenic differentiation. Removal of aldehyde groups from TEMPO-oxidized CNFs did not negatively affect cell responses. This indicates that the introduction of aldehyde groups (280 ± 14 μmol/g) through TEMPO-mediated oxidation to the surface of CNFs might have little or no effect on attachment, proliferation, and osteogenic differentiation of rat MSCs. Compared to TEMPO-oxidized and postoxidized groups, replacement of aldehyde groups with alcohols via reduction treatment decreased carboxylic groups and increased hydroxyl groups onto the surface of TO-R-CNF samples. This was beneficial not only for cell adhesion and spreading but also for the osteogenic differentiation of the cells. It is concluded that TO-R-CNF coating can have a promising potential to improve the biological performance of scaffolds for bone tissue engineering.
